# Prevalence and factors associated with preoperative anxiety in children aged 5-12 years**^1^**

**DOI:** 10.1590/1518-8345.0723.2708

**Published:** 2016-06-14

**Authors:** Louise Amália de Moura, Iohanna Maria Guimarães Dias, Lilian Varanda Pereira

**Affiliations:** 2 MSc, Assistant Professor, Faculdade de Enfermagem, Universidade Federal de Goiás, Goiânia, GO, Brazil.; 3 Undergraduate student in Nursing, Faculdade de Enfermagem, Universidade Federal de Goiás, Goiânia, GO, Brazil.; 4 PhD, Adjunct Professor, Faculdade de Enfermagem, Universidade Federal de Goiás, Goiânia, GO, Brazil.

**Keywords:** Anxiety, Child, Child, Preschool, Preoperative Period, Ambulatory Surgical Procedures, Pediatric Nursing

## Abstract

**Objective:**

to estimate the prevalence and factors associated with preoperative anxiety in children who wait for outpatient surgery.

**Method:**

cross-sectional analysis of baseline data of a prospective cohort study that investigates the predictors of postoperative pain in children aged 5-12 years submitted to inguinal and umbilical hernia repair. It was selected 210 children, which were interviewed in the preoperative holding area of a general hospital. Anxiety was evaluated using the modified Yale Preoperative Anxiety Scale (mYPAS). Sociodemographic and clinical variables were analyzed as exposure and anxiety (mYPAS final score>30) as outcome. Logistic regression was used to identify factors associated with preoperative anxiety.

**Results:**

forty-two percent (42.0%) of children presented preoperative anxiety (CI95%: 35.7%-48.6%), with mean scores equal to 30.1 (SD=8.4). Factors associated with preoperative anxiety were: age group of 5-6 years (OR=2.28; p=0.007) and socioeconomic status classified as class C (OR=2.39; p=0.016).

**Conclusion:**

the evaluation of children who wait for outpatient surgery should be multidimensional and comprise information on age and socioeconomic status, in order to help in the identification and early treatment of preoperative anxiety.

## Introduction

Anxiety is a common feeling among children in the preoperative period^1^ ^-^ ^4^. As acute stress source, anxiety induces functional changes in the central nervous system, increases the deleterious effects on the child's body when associated with other perioperative stressors^5^, produces negative behaviors^4^ ^,^ ^6^ ^-^ ^8^ and high pain intensity scores in the postoperative period^1^ ^,^ ^4^ ^,^ ^9^. In addition, anxiety causes sleep disruption, nausea, fatigue, and inadequate responses to anesthesia and analgesia^1^ ^,^ ^9^, leading to higher costs for the health services and family.

In the immediate preoperative period, which corresponds to 24 hours before surgery, discomfort is imminent for the children and their family, regardless of the type of surgery, outpatient or hospital approach and cultural context in which the child is inserted^10^. In addition, the susceptibility of the child, lack of understanding about the surgical procedure, unknown hospital environment, fear of physical injury, separation from their parents^11^ and feelings of sadness and punishment related to the fact that surgery is a scheduled procedure may contribute to such discomfort^12^. 

Several evidence indicate age^2^ ^-^ ^3^ ^,^ ^13^ ^-^ ^15^ and temperament^3^ of the child, behavioral problems during health care^15^, previous surgery and hospitalizations^4^ ^,^ ^15^, level of parental education and maternal anxiety^1^ ^,^ ^3^ ^,^ ^16^ as factors associated with preoperative anxiety in children. 

In the case of outpatient surgery, however, there are still knowledge gaps on the prevalence and factors associated with this psychological phenomenon in children. This possibly because parents and children remain together for a short period in the hospital setting and there is little availability of health professionals to provide an individualized and comprehensive care, including the multidimensional evaluation of the child in the preoperative period. It has been emphasized that the identification of children at risk might promote the use of preventive strategies and avoid problems caused in the postoperative recovery when anxiety remains at unacceptable levels. The aim of this study was to estimate the prevalence and factors associated with preoperative anxiety in children who wait for outpatient surgery.

## Methodology

This is a cross-sectional analysis of baseline data of a prospective cohort and open study, developed in Goiania, Brazil. Children were hospitalized for elective outpatient surgery, from April/2013 to February/2014. 

## Participants

It was eligible for this study children of both sexes, aged 5-12 years, with indication of elective surgeries for umbilical and inguinal hernia repair, of outpatient basis (maximum of 24 hours of hospital stay), single-port (less than two-hours procedure) and ASA I and II for operative risk. According to the scale of the American Society of Anesthesiologists (American Society of Anesthesiology) (ASA) (www.asahq.org/clinical/physicalstatus.htm), the classification of the physical status considers healthy patients as ASA I, and patients with mild to moderate systemic disease without functional limitation as ASA II.

Exclusion criteria for children were: be forwarded directly to the surgical room, which prevented preoperative contact; need to stay in the hospital for more than 24 hours, mischaracterizing ambulatory surgical care; make use of anxiolytic drugs at preoperative period; and non-attendance for surgery as scheduled. At the end of 10 months, 210 children were included in the sample. 

## Study site

Analysis of data from the Hospital Information System of SUS of Ministry of Health, in 2012, showed that nine^9^ hospitals in the city of Goiania performed pediatric ambulatory surgeries (n=291). Among children aged 5-12 years, 89% of the surgeries occurred in a general care hospital, 7.5% occurred in a pediatric hospital and 3.5% in other hospitals. Therefore, it was decided to select for this study, data of children assisted in the hospital with the highest number of visits in this municipality, and in this place, only a pediatric surgeon was the responsible for the surgical care provided.

## Data collection 

Data collection was carried out by two nurses qualified for the evaluation of anxiety, before surgery, in the preoperative holding area. Sociodemographic and economic data were collected from those responsible for the child. The assessment of anxiety and preoperative pain occurred through direct observation and child's report. The intensity of the preoperative pain was measured using a scale of faces printed. To evaluate preoperative anxiety, the observer was dressed in an ordinary outfit to avoid "anxiety" associated with white clothes. 

Study variables

### Outcome variable

- Preoperative anxiety - measured using the modified Yale Preoperative Anxiety Scale - mYPAS, translated and validated into Brazilian Portuguese^17^. 

### Exposure variables

- Socio-demographic: age of the child (5-6 years and 7-12 years); sex (male and female) and socioeconomic status (classified as class A (A1 and A2 classes), class B (B1 and B2 classes), class C (C1 and C2 classes), class D (Class D) and class E (class E), according to the Brazilian Economic Classification Criterion (CCEB)^18^. This criterion takes into account the sum score regarding the education level of the household head and the scores of objects that the family has to determine the economic class. Class A represents the highest socioeconomic status, whereas class E represents the lowest. 

- Clinics: previous surgery (yes and no), previous hospitalization (yes and no) and preoperative pain (yes and no).

### Instruments used 

Preoperative anxiety was evaluated using the modified Yale Preoperative Anxiety Scale - mYPAS^17^, observational measurement, which was planned for use in children in the immediate pre-anesthetic period and at the time of anesthetic induction. The YPAS was developed and later modified - mYPAS (Yale Preoperative Anxiety Scale modified) by Kain et al. (1997)^19^. This scale has 27 items distributed in five areas of behavior that include the child's relationship with the environment in which they are, as follows: domain 1 - activities (with 4 categories); 2 - vocalization (with 6 categories); 3 - emotional expressiveness (with 4 categories); 4 - state of awakening (with 4 categories) and 5 - interaction with family members (with 4 categories). A partial score is assigned for each domain based on the observed score, and this score is added to those of other domains, which is then multiplied by 20. The presence of anxiety is identified when the sum exceeds 30 points. The study that has adapted the mYPAS into Portuguese showed high reliability indexes (Cronbach's alpha values between 0.88 and 0.95; Spearman coefficients between 0.44 and 0.95; Kappa between 0.79 and 1.00 and Guttmann's coefficient between 0.63 and 0.90), considering the scale as reliable and reproducible^17^. 

The intensity of preoperative pain was measured using the Faces Pain Scale-Revised (FPS-R)^20^, designed for children from 4 years of age. The FPS-R is a six-point scale with faces indicating increasing intensity. The leftmost face is indicative of absence of pain, and the following faces express increasing intensity until the rightmost face, which signals great pain, enabling the child to quantify their painful experience. The psychometric properties of the FPS-R were tested and the original version has been translated into 35 languages (www.painsourcebook.ca). It has been used in many clinical trials, demonstrating the possibility of identifying pain and pain relief achieved through analgesic therapy. For this study, it was used the Brazilian Portuguese version and the score 0-2-4-6-8-10 was adopted to quantify the respective six (6) faces of the scale^21^.

### Data analysis

Here, it was decided to present the categorical variables as absolute and percentage values. The preoperative anxiety outcome was described as mean and standard deviation, with cutoff set as greater than 30 points for the overall score of the mYPAS. The prevalence of anxiety was estimated with confidence interval of 95%, and regression was used for the bivariate and multivariate analyzes. The multivariate model included variables with p≤0.10 in the bivariate analysis. All p-values less than 0.05 (p<0.05) were considered as statistically significant.

## Results

Among the 229 children scheduled for outpatient surgery, 19 (8.2%) were excluded from the study: nine (9) because they were forwarded directly to the operating room and 10 due to non-attendance in the day of surgery. Thus, the final sample consisted of 210 children. 

There was a prevalence of male children, aged 7-12 years and belonging to the socioeconomic status previously described and classified as Class C. Most of them had not experienced previous hospitalization or surgery and waited for inguinal hernia repair (Table 1).

In the preoperative holding area, 11.4% of children reported pain at the site of the hernia to be repaired, with an average intensity score equal to 4.25 (SD=2.5). 

**Table 1:** Distribution of children, according to sociodemographic and clinical variables. Goiania, GO, Brazil, 2013-2014

Variables		Children (n= 210)	
		n	%
Gender			
	Female	100	47.6
	Male	110	52.4
Age group			
	5-6 years	87	41.4
	7-12 years	123	58.6
Socioeconomic status			
	Class B	45	21.5
	Class C	125	59.5
	Class D	40	19.0
Previous hospitalization			
	Yes	93	44.3
	No	117	55.7
Previous surgery			
	Yes	30	14.3
	No	180	85.7
Preoperative pain			
	Yes	24	11.4
	No	186	88.6
Surgery type			
	Inguinal hernia repair	145	69.0
	Umbilical hernia repair	65	31.0

It was observed a prevalence of preoperative anxiety of 42.0% (CI95%: 35.7%-48.6%), with average anxiety score of 30.1 (SD=8.4), according to the mYPAS.

In the bivariate analysis, the factors associated with preoperative anxiety included the age group 5-6 years (OR=2.16) and socioeconomic class C (OR=2.27) (Table 2).

**Table 2:** Potential factors associated with preoperative anxiety, according to sociodemographic and clinical characteristics of children. Goiania, GO, Brazil, 2013-2014

Sociodemographic and clinical characteristics		Preoperative anxiety		β*	OR**^†^**	CI(95%)**^‡^**	p**^§^**
		N	%				
Gender							
	Female	41	46.6	-0.07	0.93	0.53-1.61	0.800
	Male	47	53.4				
Age group							
	5-6 years	46	52.3	0.77	2.16	1.23-3.79	0.007
	7-12 years	42	47.7				
Socioeconomic status							
	Class B	26	29.6				
	Class C	47	53.4	0.82	2.27	1.13-4.54	0.020
	Class D	15	17.0	0.82	2.28	0.95-5.45	0.064
Previous hospitalization							
	Yes	44	50.0	0.39	1.49	0.85-2.59	0.158
	No	44	50.0				
Previous surgery							
	Yes	16	18.2	-0.53	0.58	0.26-1.26	0.174
	No	72	81.8				
Preoperative pain							
	Yes	10	11.4	-0.01	0.98	0.41-2.34	0.980
	No	78	88.6				

*Beta coefficient

†*Odds Ratio*

‡Confidence interval of 95%

§Significance level

After adjusting, gender, age group of 5-6 years and socioeconomic class C remained associated with preoperative anxiety. Children with these characteristics were twice as likely to present preoperative anxiety (Table 3).

**Table 3:** Factors associated with preoperative anxiety. Goiania, GO, Brazil, 2013-2014

Variables	β*	OR**_adjust_** **^†^**	CI(95%)**^‡^**	p**^§^**
Age group of 5-6 years	0.82	2.28	1.25-4.16	0.007
Socioeconomic class C	0.87	2.39	1.17-4.87	0.016

*Beta coefficient

†*Odds Ratio* adjusted by gender

‡Confidence interval of 95%

§Significance level

## Discussion

The results of this study demonstrate that many children who wait for outpatient surgery experience preoperative anxiety. Factors such as age and socioeconomic status influence the occurrence of this phenomenon. 

It was observed that 42.0% of children were anxious in the preoperative holding area of the hospital. Brazilian researchers estimated a high prevalence (81.6%) of anxiety among children (4.67±0.96 years) weeks prior to surgery, at the time of outpatient preoperative evaluation^22^. In the evaluation of children aged 4-8 years, prevalence rates of 38.9%^23^ and 84.0%^24^ were found in the preoperative holding area. 

Before surgery, the child tends to understand this event as a threat that, in just few minutes, causes different feelings^12^. Contradictory prevalence of preoperative anxiety may be related to the age of the children^22^ ^-^ ^24^, anxiety measurement instrument^19^, lack of information about the surgery to be performed, separation from their parents^10^ and previous experience in health care^3^ ^-^ ^4^.

In this study, for example, after discharge from the post-anesthetic recovery room, the children returned to the same preoperative holding area. It is very likely that those waiting for the time to enter the operating room tended to express higher levels of fear and anxiety because they saw post-operated children distressed or crying. High levels of anxiety impair the recovery of children, and subsequently affect the physical and psychological health, impair the ability to deal with medical treatment and generate negative behavior regarding future health cares^1^ ^,^ ^4^ ^,^ ^7^ ^-^ ^9^ **_._**

Currently, in order to prevent the effects of this situation, preoperative preparation programs including the participation of children and their parents, before and after surgery, have been proposed^11^ ^,^ ^25^ ^-^ ^26^.

In this sense, the first move is the identification of children at risk. Age is a factor that interferes in the occurrence of anxiety in the preoperative period, a finding consistent with those from previous studies^2^ ^-^ ^3^ ^,^ ^13^ ^-^ ^15^. In the pediatric population, the perception of anxiety also depends on the developmental stage and cognitive potential of the child, since different responses can be observed among those facing the same stressor agent^27^. 

Children under the age of seven years (pre-school), for example, are able to correlate anxiety with physical symptoms^27^. Faced with an imminent surgical procedure, they seek explanations for the situation since they have fears about the surgery^26^. As for the older children (students), with more advanced cognitive development, they can get involved in decision-making and their feeling of fear is certainly related to the possibility of being unable to recover from the anesthesia^26^. Therefore, the child should be treated and understood individually, taking into account the development stage in which they are, which represents a challenge for professionals and parents who experience the situation. Further researches on preoperative anxiety in children at different stages of the development are desirable.

Regarding the socioeconomic status, evidences reinforce the relationship of this variable with preoperative anxiety in children. However, among the studies found^15^ ^-^ ^16^, in which anxiety was assessed during induction of anesthesia, these associations were not significant. 

It is understood that the socioeconomic status may reflect on different physical and psychological conditions among children and hence lead to an ineffective coping with new situations, such as surgery. Furthermore, most children participating in this research belong to the socioeconomic class C and were assisted at a public hospital, where the demand for services and rates of procedures/day/professional are high. This may reduce the supply of individualized care in the preoperative period and hinder the identification of specific health care needs.

Nurses are professionals able to effectively influence the experience of the children^28^ and parents^29^ in the perioperative environment. It is their responsibility the multidimensional evaluation of the child during their routine work^30^, since the psychological, social and economic variables might interfere with proper surgical recovery^31^. 

In this sense, this study aims to contribute to the advancement of knowledge about anxiety in the period before a pediatric outpatient surgery, emphasizing the need for investigations that include the evaluation of this phenomenon throughout the perioperative period. Among its limitations, it is worth mentioning the lack of assessment of parental anxiety, since the presence of anxiety in the child may be related to the high levels of maternal anxiety^1^ ^,^ ^15^ ^-^ ^16^ ^,^ ^25^ ^-^ ^26^. 

## Conclusion

The high proportion of children who wait for outpatient surgery experience preoperative anxiety. Age and socioeconomic status influence the occurrence of this phenomenon. 

Such findings indicate the need for assessment using a biopsychosocial approach to children, aiming at the proper management of anxiety in the preoperative period, early recovery and reduction of postoperative problems.
